# Short-Term and Long-Term Effects of Off-Job Activities on Recovery and Sleep: A Two-Wave Panel Study among Health Care Employees

**DOI:** 10.3390/ijerph15092044

**Published:** 2018-09-18

**Authors:** Jan de Jonge, Akihito Shimazu, Maureen Dollard

**Affiliations:** 1 Human Performance Management Group, Eindhoven University of Technology, P.O. Box 513, 5600 MB Eindhoven, The Netherlands; 2 School of Psychology, Asia Pacific Centre for Work Health and Safety, University of South Australia, P.O. Box 2471, Adelaide 5001, Australia; ashimazu-tky@umin.ac.jp (A.S.); maureen.dollard@unisa.edu.au (M.D.); 3 Center for Human and Social Sciences College of Liberal Arts and Sciences, Kitasato University, 1-15-1 Kitasato, Minami-ku, Sagamihara, Kanagawa 252-0373, Japan

**Keywords:** recovery from work, off-job recovery activities, panel study, health care employees

## Abstract

This study examined whether particular recovery activities after work have a positive or negative effect on employee recovery from work (i.e., cognitive, emotional, and physical detachment) and sleep quality. We used a two-wave panel study of 230 health care employees which enabled looking at both short-term and long-term effects (i.e., two-year time interval). Gender, age, marital status, children at home, education level, management position, and working hours were used as control variables. Hierarchical multiple regression analyses showed that work-related off-job activities were negatively associated with cognitive and emotional detachment in both the short and long run, whereas low-effort off-job activities were positively related to cognitive detachment in the short run. Moreover, household/care off-job activities were positively related to sleep quality in the long run, whereas physical off-job activities were negatively associated with sleep quality in the long run. The long-term findings existed beyond the strong effects of baseline detachment and sleep quality. This study highlights the importance of off-job recovery activities for health care employees’ detachment from work and sleep quality. Practical implications and avenues for further research are discussed.

## 1. Introduction

Health care employees are facing increasingly high demands at work, which may cause job strain and burnout [1], and even reduced work performance [2]. While there is mounting evidence on the consequences of high job demands, less attention has been paid to the role of off-job recovery from job strain and the opportunity to engage in different types of off-job recovery activities [3]. Examples of the latter are household and care activities (e.g., child), social activities, physical activities, and even work-related activities. However, not all these activities might be equally beneficial for off-job recovery. Empirical research had shown that some types could be harmful, such as work-related activities, whereas activities such as social and physical activities may enhance off-job recovery (for overviews, see [4,5]). For instance, a diary study by Oerlemans and his team [3] showed that work-related and household activities were negatively associated with recovery at bedtime, particularly in the case of low happiness during those activities. However, it should be noted that overall empirical evidence is mixed, and the jury is still out on this issue. For this reason, the present study examined whether recovery activities pursued during off-job leisure time have a positive or negative effect on employee recovery from work and sleep quality. We used a longitudinal design which facilitated looking at short-term and long-term effects.

Recovery can generally be defined as a process of unwinding and restoration during which an employee’s functioning and strain level returns to its pre-stressor level [3,5]. Thus, recovery can be considered as a process opposite to the strain process, in which detrimental effects of stressful situations are at least alleviated or even eliminated. If recovery is successful, employee health and performance improve. If not, health and performance will be affected, and the employee starts the next working day in a suboptimal state [6].

The process of recovery can be explained by two theoretical frameworks; that is, the Effort-Recovery (E-R) Model [7] and the Conservation of Resources (COR) Theory [8]. These frameworks generally assume that an employee’s reservoir of personal resources (e.g., concentration, focus, resilience) may be depleted at the end of a working day, which makes recovery necessary. They propose that successful recovery can be reached in the following ways. First, according to the E-R Model, recovery usually occurs when job demands end. Employees are then able to replenish their personal resources and detach from work during off-job time. Second, recovery can be obtained by accumulating additional resources during leisure time such as learning new skills [8]. Consequently, employees are better able to deal with future job demands. Finally, off-job activities can contribute to recovery if they help employees to replenish resources.

Sonnentag and Geurts [9] suggested making a distinction between recovery as a process and recovery as an outcome. Recovery as a process refers to the activities and experiences that may lead to a change in functioning and strain level. Recovery as an outcome implies an employee’s psychophysiological state that is reached after a recovery period. As Demerouti and associates [4] mentioned, future research should take into account the recovery experiences that employees derive from their off-job recovery activities, we will focus on recovery as a process; that is, both recovery activities and recovery experiences.

In line with Ten Brummelhuis and Trougakos [10], off-job recovery activities can be split into two main categories; that is, *high-duty* activities such as work-related and household/care tasks, and *leisure* activities such as social, physical, creative, and low-effort activities. Demerouti and her team [4], as well as Sonnentag and colleagues [5], summarized existing empirical research regarding these main categories. The overall picture emerging from these overviews is that high-duty activities potentially hinder off-job recovery (e.g., [3]) and that leisure activities are potentially promoting off-job recovery (e.g., [10]). We added another leisure activity, i.e., a daytime nap, which is commonly referred to as a short sleep lasting from a few minutes to a few hours [11,12]. Especially short naps (less than 30 min a day) can reduce sleepiness, help to restore daytime arousal levels, improve cognitive functioning and emotional state, and enhance performance. So, naps of brief duration can be beneficial to employees and can have an important restorative function [11,12].

The general idea behind this is that work-related tasks keep employees in a working mode and impede detachment, while leisure activities provide more opportunities for reducing job strain and replenishing resources. In addition, given its restorative function, we expect that taking a daytime nap after work will also promote off-job recovery.

With regard to recovery experiences, Sonnentag and Fritz [13] came up with different types of experiences, of which *psychological detachment* is most salient. Psychological detachment refers to the personal experience of leaving work behind, to switch off completely, and to forget about work during off-job time. Beneficial effects of detachment have been widely reported in the literature, e.g., [5]. For instance, cross-sectional studies showed that employees who fully detach from work report lower levels of job strain and higher levels of well-being (e.g., [6,13]). Daily survey studies suggest that detachment might be particularly important after stressful working days (e.g., [3,10]).

So far, most studies on off-job recovery have not included the important role of sleep [4]. Sleep seems to be essential to complete the recovery process effectively [4]. Off-job recovery activities that promote off-job recovery should improve sleep quality [9]. On the other hand, activities that hinder off-job recovery should increase sleep problems. For example, a study by Cropley and associates [14] showed that the inability to stop thinking about work issues during off-job time was associated with more sleep problems. To conclude, sleep problems can be considered outcomes of longer-term insufficient recovery [4,9,14,15].

Finally, much research on off-job recovery draws on cross-sectional or daily-diary designs [4,5]. Although these types of research design are imperative for mapping day-to-day variations in daily recovery experiences and sleep, it is difficult to connect these day-to-day variations to longer-term recovery experiences and sleep. For this reason, it would be interesting to see how day-to-day off-job activities contribute to longer-term recovery experiences and sleep. Some effects usually evolve during a much longer timeframe, and it is difficult to capture these periods using a cross-sectional or daily-diary design. Sonnentag et al. [5] noticed that we know surprisingly little about how the recovery process unfolds over a longer timeframe. Based on the daily equilibrium assumption of our two theoretical frameworks, we expect that short-term and long-term relations between (1) off-job activities and (2) recovery experiences and sleep are stable.

Hence, three key hypotheses guided our study:

**Hypothesis** **1** **(H1).** *Off-job leisure time spent on high-duty activities (e.g., work-related activities, household/care activities) would be negatively associated with off-job recovery and sleep quality.*

**Hypothesis** **2** **(H2).** *Off-job leisure time spent on leisure activities (e.g., social, creative, physical, and low-effort activities, taking a nap) would be positively associated with off-job recovery and sleep quality.*

**Hypothesis** **3** **(H3).** *Short-term (i.e., cross-sectional) and long-term (i.e., two-wave, two-year panel) associations between (1) off-job activities and (2) off-job recovery and sleep quality are stable.*

## 2. Materials and Methods

### 2.1. Study Design, Data, and Procedure

We conducted a two-wave panel design with a two-year time interval in three general hospitals in the Netherlands. All health care employees (e.g., nurses, doctors, laboratory staff) received an email with a unique link to an online survey, which was linked to their email addresses for second-round identification. At Time 1, 556 employees received the questionnaire, and 395 people returned it (71% response rate). At Time 2, 368 out of 541 employees returned the questionnaire (68% response rate). The final panel sample reflects those who participated at both times, and consisted of 230 participants (41% response rate). A breakdown of the demographic characteristics showed that 83.4% of the participants were female. The mean age of the group was 45.7 years (SD = 10.7; range 23–63 years). The majority of the employees had finished higher vocational education (60.1%). Most of the respondents were married or lived together (76.9%), and 49.9% had children at home. The percentage of management positions was 6.4%. Finally, 26.6% of the employees worked on a full-time basis (i.e., at least 36 h per week), and mean working time was 29.8 h per week (SD = 6.3).

All subjects gave their informed consent for inclusion before they participated in the study. The study was conducted in accordance with the Declaration of Helsinki, and the protocol was approved by the Medical Ethics Committee of the UMC St. Radboud in the Netherlands (Project identification code: 2012/546).

### 2.2. Variables and Instruments

Off-job activities were measured as either high-duty activities or leisure activities, based upon the work of Sonnentag and colleagues [5,16]. The general question was: “How much time do you spend on average after a shift on the following activities?” High-duty activities to be answered were work-related activities and household and/or (child) care activities. Leisure activities to be answered were social activities (e.g., visits, telephone conversations), physical activities (e.g., sports, cycling, walking), creative activities (e.g., music, painting), low-effort activities (e.g., reading, watching TV), and taking a nap. All items were rated on a 5-point frequency scale: 1 (0 h), 2 (0–1 h), 3 (1–2 h), 4 (2–3 h), and 5 (more than 3 h). Test-retest reliabilities (i.e., stabilities) of single items with a two-year time interval ranged from 0.30 to 0.56 (*p* < 0.001).

We measured off-job recovery using the DISQ-R, a well-validated scale developed by De Jonge and colleagues [6]. This scale consists of three recovery experiences, i.e., cognitive, emotional, and physical detachment after work. Each component was measured with three items, which were rated on a 5-point frequency scale ranging from 1 (never) to 5 (always). Examples of items are, “After work, I put all thoughts of work aside” (cognitive; Time 1 *α* = 0.74; Time 2 *α* = 0.79; stability = 0.67, *p* < 0.001), “After work, I emotionally distance myself from work” (emotional; Time 1 *α* = 0.73; Time 2 *α* = 0.76; stability = 0.61, *p* < 0.001), and “After work, I shake off the physical exertion from work” (physical; Time 1 *α* = 0.59; Time 2 *α* = 0.67; stability = 0.49, *p* < 0.001).

Sleep quality was measured by three items derived from the Maastricht Questionnaire [17]. For instance, “Do you often have problems falling asleep?” (reverse coded). The possible responses are 1 (no), 2 (sometimes), and 3 (yes). Time 1 *α* = 0.61; Time 2 *α* = 0.61; stability = 0.67, *p* < 0.001.

We used gender, age, marital status, children at home, education level, management position, and working hours as control variables.

### 2.3. Statistical Analysis

First, Pearson zero-order correlational analyses were conducted to obtain an initial overview of the data. Second, hierarchical multiple regression analyses (HMRAs) were used to examine the relation between off-job activities and the four outcome measures (i.e., cognitive, emotional, and physical detachment as well as sleep quality). All analyses were performed in IBM SPSS Statistics 25. No significant violations of linear regression assumptions were detected. The HMRAs were conducted with simultaneous entry of variables within each hierarchical step. We performed two hierarchical modeling steps accordingly. For the cross-sectional analyses, Time 1 control variables were entered in Step 1, because prior research has indicated that these variables may affect our outcome measures (e.g., [3,5,10]). In Step 2, we added all Time 1 off-job activities (i.e., high-duty and leisure activities) as independent variables. In case of the panel analyses, the dependent variable at Time 1 was entered in Step 1 as a covariate which is in line with longitudinal statistical testing. In Step 2, Time 1 control variables were entered. Finally, in Step 3, we added all Time 1 off-job activities as independent variables. We presented standardized beta-weights and their significance for individual predictor variables in Tables 2 and 3, as well as the overall explained variance (R^2^) of the final regression model.

## 3. Results

Table 1 reports the zero-order correlations of the study variables. Apart from gender and marital status, all control variables were significantly related to our variables of interest. Gender and marital status were significantly related to several other control variables. For reasons of comparison with other studies, we have decided to keep them all in the HMRAs.

### 3.1. Cross-Sectional Analyses

The results of the cross-sectional HMRAs are depicted in Table 2. Looking at the overall model test, it appears that only the regression model for cognitive detachment was significant (F(14,160) = 3.03; *p* < 0.001). A closer look at the beta-weights of the individual predictor variables shows that work-related off-job activities were negatively associated with cognitive detachment (β = −0.26; *p* < 0.01). This was in line with Hypothesis 1. In other words, health care workers who were involved in more work-related off-job activities also reported being less able to cognitively detach after work. The same is true for emotional detachment (β = −0.18; *p* < 0.05), but in this case the overall regression model was not significant. Furthermore, there is a positive relation (β = 0.18; *p* < 0.05) between low-effort activities and cognitive detachment (in line with Hypothesis 2). Staff who were involved in low-effort activities reported being better able to cognitively detach during non-work time. The explained variance (R^2^) of the final regression models ranged from 0.07 to 0.23.

### 3.2. Panel Analyses

Table 3 shows the findings of the longitudinal HMRAs (two-year time interval). In this case, the dependent variable at Time 1 was first entered into the model in order to model change in the respective outcome. All four regression models were significant, with R^2^s ranging from 0.29 to 0.55. As far as cognitive detachment is concerned, again work-related off-job activities were negatively associated with this outcome measure, but now over time (β = −0.24; *p* < 0.001). More specifically, health care staff who were involved in more work-related off-job activities reported also to be less able to cognitively detach after work in the long run. Results for emotional detachment also show a negative lagged relation between work-related off-job activities and this outcome measure (β = −0.15; *p* < 0.05). Both lagged findings were in line with Hypothesis 1.

Despite the overall model significance, there were no significant predictors for physical detachment in the long run. Finally, lagged results for sleep quality show two significant predictor variables: Household/care and physical off-job activities. Health care employees who were involved in more household/care off-job activities reported higher sleep quality in the long run (β = 0.14; *p* < 0.05). In contrast, staff who were involved in more physical off-job activities reported lower sleep quality in the long run (β = −0.16; *p* < 0.01). However, these findings were not in line with Hypotheses 1 and 2.

### 3.3. Short-Term and Long-Term Findings

If we look at the stability of both cross-sectional and panel results (Hypothesis 3), it appears that only the findings of work-related off-job activities were stable over time. This was particularly true for cognitive detachment, and to a lesser extent for emotional detachment.

## 4. Discussion

This study examined whether particular off-job recovery activities have a positive or negative effect on employee recovery from work and sleep quality. We used a two-wave panel design which enabled looking at both short-term and long-term effects. In addition, it allowed a more rigorous interpretation of causality than a cross-sectional design only.

Analyses showed that work-related activities were negatively associated with cognitive and emotional detachment in both the short and long run, whereas low-effort activities were positively related to cognitive detachment in the short run only. Moreover, household/care activities were positively related to sleep quality in the long run. Contrarily, physical activities were negatively associated with sleep quality in the long run. The long-term findings existed beyond the strong effects of baseline detachment and sleep quality. So, we found supporting evidence for our three hypotheses except for sleep quality. We will discuss these findings in more detail below.

### 4.1. Theoretical and Practical Implications

This study reveals that work-related activities were negatively related to cognitive and emotional detachment from work in the short and long run. Therefore, employees seem to continuously draw on identical resources as those needed during working hours [16,18]. This can empty an employee’s resource reservoir and, hence, increase strain [4,8]. In other words, continuous thinking of work and conducting work-related activities might impede the necessary recovery processes, and detachment from one’s job cannot take place [7,16]. Our findings are in line with other empirical studies. For instance, a diary study of four lagged days among 74 Dutch nurses showed that work-related off-job activities were negatively related to feeling recovered on the next morning [10].

We also found that household/care activities were unrelated to detachment, which is in line with most other research studies (e.g., [3,10,19]). Interestingly, they were positively associated with sleep quality in the long run. An explanation could be that this is a very broad category which entails off-job activities that are resource-depleting as well as activities that are resource-enhancing [5]. In some cases, household/care activities may be experienced as positive which help to disengage from job strain and, hence, increase sleep quality. Another explanation is that there are personal differences suggesting that some people experience these as positive and others as negative [4].

With regard to the leisure activities measured, two out of five off-job activities showed a significant effect: (1) low-effort activities were positively related to cognitive detachment in the short run, and (2) physical activities were negatively associated with sleep quality in the long run. As its label implies, low-effort activities such as watching TV or listening to music require hardly any effort and consequently have no further impact on resource-depletion. In addition, these activities usually do not pose any demand on the same resources required for job demands. This makes them almost perfect off-job recovery activities. However, empirical support in the literature is mixed [5], and was sought to be explained by the role of moderator variables such as motivation [10] or affective predispositions [20]. Indeed, research by Ten Brummelhuis and Trougakos [10] showed that the positive association between low-effort activities and morning recovery was stronger when nurses had high intrinsic motivation for these activities. Finally, contrary to predictions, we did not find any evidence for the presumed restorative function of taking a nap, not in the short-term and not in the long-term. One explanation could be that we include neither the time length of the nap nor the time naps are taken during the day. Research has shown that only short naps are able to restore daytime arousal levels and to improve cognitive functioning and emotional state. Moreover, naps taken during the post-lunch dip seem to have a greater restorative function than naps taken in the morning or evening [11,12]. Generally, frequent and longer naps may lead to adverse health effects in the long run [11].

The inhibiting role of physical activities on sleep quality is interesting. We assume that most of our respondents are involved in sports, cycling, and walking after their shifts. Research has only indicated that the combined effects of late-day exercise—including increased heart rate, higher body temperature, and adrenaline/cortisol boost—are a bad recipe when it comes to getting a good night’s sleep [21]. However, a laboratory study among eleven physically fit young adults showed that late-night vigorous exercise did not disturb sleep quality [22]. A large empirical survey study conducted by the US National Sleep Foundation [21] among 1000 participants found no major differences in sleep quality between those who had performed vigorous and/or moderate exercises within four hours before bedtime. Only three percent of late-day exercisers said they slept worse on days when they exercised, compared to days when they did not exercise at all. So, there are people who won’t have any problems with a late-day workout and sleep. However, if someone already has trouble falling asleep, late-day physical exercise may worsen the problem [21,22]. A key difference between most empirical studies and our study is that we measured the change in sleep quality over two years in our longitudinal regression analyses. Our findings preliminarily indicate that for some people after-work exercising may lead to increased arousal and sustained activation of adrenaline/cortisol levels, which may also affect sleep quality in the long run.

The current findings have implications for practice as well. Our results shed more light on those off-job recovery activities that facilitate or hamper off-job recovery and sleep quality in the short and long run. First, health care employees seem to detach most when they are not engaged in work-related activities during off-job leisure time. Modern technologies such as smartphones and tablets as well as social media imply that employees stay tuned to work while having leisure time. This could create a 24/7 availability for work-related tasks, and makes boundaries between work and home invisible. Consequently, cognitive and emotional detachment from work cannot take place. The present study suggests that this has negative short-term and long-term effects. Management plays an important role here, too. They should create a work climate in which working beyond regular work hours is not “business as usual”, as this kind of activity impedes necessary recovery processes. Second, health care employees also seem to detach when they are pursuing low-effort off-job activities. Involvement in leisure activities such as watching TV, reading a book, listening to music, or even doing anything to ensure that job demands truly end. Employees are then able to replenish their personal resources and fully detach from work. In general, we believe that employees should spend leisure time on leisure activities that they like most. Ten Brummelhuis and Trougakos [10] showed that the recovery potential is highest in cases where leisure activities are intrinsically motivated, and people have fun doing them. Finally, our findings suggest that employees with sleep troubles should be wary of working out too close to bedtime. Adrenaline and cortisol levels are high, the brain is active, and it is difficult to wind down; and this is associated with a suboptimal recovery state the next morning. If people fall into that group and have experienced trouble sleeping after a late-day workout, it is recommended that physical exercise is minimized within three or four hours of bedtime.

### 4.2. Strengths and Limitations

This study has some strengths and limitations. A strength was the use of a cross-sectional design and a panel design which allowed investigation of both short-term and long-term effects. The panel design also overcomes causality issues that are common in the first type of design. A limitation is that common method variance due to using self-reported data may have played a role, although research studies have shown that this influence is not as high as commonly believed [23]. Nevertheless, multi-source and/or multi-method studies are recommended to deal with this kind of bias. A closely related limitation is the risk of the triviality trap [5,20]. Put differently, there is potential for content overlap between some off-job activities and off-job recovery experiences. For instance, low-effort activities and off-job detachment share low activation experiences. In line with earlier research [20], we have tried to tackle this potential overlap to operationalize both constructs in a rather different way. Third, the panel response rate was not very high, which could have created bias in our findings. However, comparison of panel members with dropouts showed no significant differences in our key variables. This indicates that non-response seemed not to systematically bias the main findings. A fourth limitation is that Cronbach’s alphas for a few measurement scales were relatively low, which may also be partly due to the panel sample size. Particularly, Time 1 physical detachment yielded a lower Cronbach’s alpha (0.59), although Time 2 alpha was adequate. In previous studies, the internal consistency of this scale has shown satisfactory to good results, e.g., [6]. For future studies, it is recommended that its number of items is increased and to reassess the psychometric properties of this scale. A final limitation is that our two-year panel interval might be too long to detect a higher number of significant findings. More waves and preferably with shorter time intervals are highly recommended.

### 4.3. Future Research

A first avenue for future research could be a refinement of our measures for off-job recovery activities. We measured broad categories of these activities rather than specific components within each category. For instance, it is recommended that the type of work-related or household/care activities are specified, as well as the type of low-effort activities, physical activities, and taking a nap. Moreover, the combined effects of specific off-job activities can be investigated, too. In this way, their full potential to facilitate detachment from work can be identified, which may also lead to refined implications for real practice.

Another avenue for future research is to further examine the particular, moderating, role of individual characteristics such as affect, motivation, and personal control in the relation between off-job activities and off-job recovery (e.g., [10,20]). It is highly likely that personal preferences for specific off-job activities are particularly beneficial for specific off-job detachment components [24]. For example, in their daily survey among 95 administrative employees, Hunter and Wu [25] showed that engagement in personally-preferred break activities helped recovery best.

A final avenue for future research is to test our hypotheses in other research areas such as remaining human services, industry, or retail trade. It would be interesting to see whether or not findings of the current study will hold for their employees, too.

## 5. Conclusions

The current study shows the important role of off-job recovery activities for health care employees’ detachment from work and sleep quality. Off-job recovery can help to replenish existing personal resources, and can even help to accumulate additional resources during leisure time. As a consequence, employees are better able to deal with future job demands. Managers should be aware that allocating job demands during leisure time may be negative for employee health and well-being, which in turn may have a destructive effect on employee performance. Both managers and employees should find creative ways to accomplish job demands within regular work hours.

## Figures and Tables

**Table 1:** Pearson zero-order correlation coefficients of the variables (*n* = 230).

Measure	1	2	3	4	5	6	7	8	9	10	11	12	13	14	15	16	17	18	19	20	21
1 Age																					
2 Gender	−0.05																				
3 Mar. status	0.18 *	−0.12																			
4 Children	0.05	−0.01	0.37*																		
5 Education	0.11	−0.05	0.14	−0.08																	
6 Work time	−0.11	−0.40 **	−0.20 *	−0.36 **	0.20 *																
7 Job level	0.09	0.06	0.07	−0.01	0.18 *	0.22 **															
8 Work-rel.	−0.10	−0.01	−0.02	0.05	0.13	0.23 **	0.10														
9 Household	0.00	0.14	0.13	0.39 **	−0.01	−0.26 **	−0.19 *	0.25 **													
10 Social	−0.20 *	0.09	−0.15	−0.09	0.05	0.05	−0.14	0.19 *	0.32 **												
11 Physical	−0.04	−0.08	0.01	−0.07	0.06	0.13	0.05	0.11	0.11	0.29 **											
12 Creative	0.30 **	−0.05	0.05	−0.05	0.12	0.03	−0.02	0.11	0.10	0.24 **	0.31 **										
13 Low−effort	−0.12	0.02	−0.06	−0.13	0.06	−0.03	−0.09	−0.04	0.04	0.18 *	0.23 **	0.18 *									
14 Nap	−0.10	−0.01	0.01	−0.07	−0.00	0.07	−0.09	0.03	−0.06	−0.12	−0.04	0.04	0.08								
15 Cogdet T1	−0.04	0.10	−0.01	0.04	−0.30 **	−0.25 **	−0.12	−0.32 **	−0.01	−0.01	−0.00	−0.05	0.17 *	−0.06							
16 Emodet T1	−0.13	0.13	−0.05	0.01	−0.13	−0.18 *	−0.12	−0.23 **	−0.05	0.11	−0.01	−0.02	0.10	−0.08	0.67 **						
17 Phydet T1	−0.05	0.03	−0.05	−0.04	0.03	0.04	0.08	−0.07	−0.15	0.05	−0.03	−0.04	−0.05	−0.13	0.28 **	0.40 **					
18 Sleep T1	0.03	0.07	−0.05	−0.05	0.00	−0.04	−0.12	0.14	0.04	−0.06	−0.03	−0.05	−0.04	0.10	−0.24 **	−0.23 **	−0.13				
19 Cogdet T2	−0.03	0.01	−0.06	−0.04	−0.26 **	−0.17 *	−0.28 **	−0.40 **	0.00	0.06	0.01	−0.03	0.15	−0.07	0.67 **	0.55 **	0.15	−0.17 *			
20 Emodet T2	−0.22 **	−0.02	−0.02	−0.06	0.08	−0.00	−0.16 *	−0.24 **	−0.01	0.06	−0.00	−0.05	0.09	−0.03	0.56 **	0.61 **	0.22 **	−0.19 *	0.74 **		
21 Phydet T2	−0.02	−0.07	−0.00	−0.02	−0.05	0.11	0.15	0.03	−0.06	0.06	0.11	0.09	−0.07	−0.09	0.13	0.17 *	0.49 **	−0.19 *	0.10	0.20 *	
22 Sleep T2	0.09	0.14	−0.12	−0.16 *	0.03	0.02	−0.06	0.03	−0.08	0.01	0.13	−0.02	0.05	−0.04	−0.22 **	−0.18 *	−0.04	0.67 **	−0.23 **	−0.27 **	−0.24 **

* significant at *p* < 0.05; ** significant at *p* < 0.01; Mar. = Marital; rel. = related; T1 = Time 1; T2 = Time 2; Cog = Cognitive; Emo = Emotional; Phy = Physical; det = detachment.

**Table 2:** Hierarchical regression analyses: Cross-sectional results (*n* = 230).

Predictor Variables Time 1	Cognitive Detachment Time 1	Emotional Detachment Time 1	Physical Detachment Time 1	Sleep Quality Time 1
Control Variables:				
Age	−0.01	−0.10	−0.06	−0.05
Gender	−0.01	0.06	0.02	−0.10
Marital status	−0.01	−0.07	0.01	0.01
Children at home	−0.04	0.06	0.04	0.12
Education	−0.21 **	−0.04	0.03	0.02
Working time	−0.14 **	−0.11	−0.01	−0.02
Job level	−0.04	−0.05	0.05	0.14
High-duty Off-job Activities:				
Work-related	−0.26 **	−0.18 *	−0.07	−0.16
Household/care	−0.02	−0.11	−0.19	−0.05
Leisure Off-job Activities:				
Social	0.04	0.14	0.15	0.14
Physical	0.03	−0.01	0.00	−0.02
Creative	−0.04	0.02	−0.04	0.04
Low-effort	0.18 *	0.06	−0.07	0.03
Nap taking	−0.06	−0.07	−0.09	−0.08
Model Test	F(14,160) = 3.03 *** R^2^ = 0.23	F(14,159) = 1.37 R^2^ = 0.12	F(14,156) = 0.76 R^2^ = 0.07	F(14,160) = 0.85 R^2^ = 0.08

* significant at *p* < 0.05; ** significant at *p* < 0.01; *** significant at *p* < 0.001.

**Table 3:** Hierarchical regression analyses: Panel results (*n* = 230).

Predictor Variables Time 1	Cognitive Detachment Time 2	Emotional Detachment Time 2	Physical Detachment Time 2	Sleep Quality Time 2
Time 1 Dependent	0.54 ***	0.59 ***	0.48 ***	0.69 ***
Control Variables:				
Age	−0.02	−0.18 *	0.00	−0.12
Gender	−0.05	−0.07	−0.04	−0.12
Marital status	−0.03	0.06	0.02	0.00
Children at home	−0.04	−0.09	0.02	0.06
Education	−0.04	−0.04	−0.13	−0.01
Working time	0.02	0.10	0.14	−0.03
Job level	−0.14 *	−0.05	0.10	0.06
High-duty Off-job Activities:				
Work-related	−0.24 ***	−0.15 *	0.02	0.03
Household/care	0.07	0.12	0.04	0.14 *
Leisure Off-job Activities:				
Social	0.05	−0.05	0.00	−0.05
Physical	0.03	−0.01	0.07	−0.16 **
Creative	−0.02	0.03	0.09	0.08
Low-effort	−0.02	−0.01	−0.04	−0.06
Nap taking	−0.03	−0.02	−0.03	0.10
Model Test	F(15,157) = 8.76 *** R^2^ = 0.48	F(15,157) = 7.40 *** R^2^ = 0.44	F(15,153) = 3.78 *** R^2^ = 0.29	F(15,158) = 11.85 *** R^2^ = 0.55

* significant at *p* < 0.05; ** significant at *p* < 0.01; *** significant at *p* < 0.001.
